# The effect of social support on physical activity of hemodialysis patients : the chain mediating role of exercise self-efficacy and barriers to exercise benefits

**DOI:** 10.1186/s40359-026-05261-3

**Published:** 2026-08-11

**Authors:** Mei Huang, Siran Zhao, Jie Gao, Honghong Lv, Ya Zhang, Yanqing Kang, Xinyue Xu, Aili Lv, Chunping Ni

**Affiliations:** 1 School of Nursing, Air Force Medical University, Xi’an, Shaanxi China; 2 Department of Nephrology, Second Affiliated Hospital of Air Force Medical University, Xi’an, Shaanxi China; 3 Health Science Center, Xi’an Jiaotong University, Xi’an, Shaanxi China

**Keywords:** Hemodialysis patients, Social support, Physical activity, Exercise self-efficacy, Barriers to exercise benefits, Self-determination theory, Mediating effect

## Abstract

**Background:**

Maintenance hemodialysis patients exhibit low physical activity, which is associated with sarcopenia, increased risk of cardiovascular events, and elevated all-cause mortality. Social support is an important resource for promoting healthy behaviors. But the mechanisms of influencing these patients’ intrinsic psychological processes to raise physical activity levels are unclear.

**Methods:**

This study was a cross-sectional observational study from September 2021 to February 2022, enrolling 460 hemodialysis patients from four tertiary hospitals in Xi’an City. We assessed social support, exercise self-efficacy, and exercise benefits and barriers perceptions with the Social Support Rating Scale (SSRS), the Self-Efficacy for Exercise Scale (SEE), and the Dialysis Patient-perceived Exercise Benefits and Barriers Scale (DPEBBS) respectively. Daily total step count was measured using an Omron electronic pedometer (model HJ-321) worn continuously for seven days. Descriptive statistics, Pearson correlation analysis, and chain mediation analysis using the PROCESS macro developed by Hayes (Model 6) were performed; the significance of mediation effects was tested via bootstrapping.

**Results:**

Pearson correlation analysis revealed significant positive correlations among social support, exercise self-efficacy, perceived exercise benefits and barriers, and total daily steps (*r* = 0.289–0.507; all *P* < 0.01). Chain mediation model analysis indicated social support’s total effect on daily steps was 0.289. Specific mediation pathways included: (1) social support → exercise self-efficacy → total daily steps; (2) social support → perceived barriers to exercise benefits → total daily steps; (3) social support → exercise self-efficacy → perceived barriers to exercise benefits → total daily steps.

Significant positive correlations were observed among social support, exercise self-efficacy, perceived exercise benefits and barriers, and total daily steps (all *P* < 0.01). Chain mediation analysis revealed that social support’s total effect on daily steps was 0.289 (95% CI: 0.201–0.377). Three significant mediation pathways were identified: social support → exercise self-efficacy → daily steps; social support → perceived barriers to exercise benefits → daily steps; and the chain mediation pathway social support → exercise self-efficacy → perceived barriers to exercise benefits → daily steps.

**Conclusion:**

Social support not only directly and positively influences physical activity levels among patients undergoing hemodialysis but also indirectly promotes physical activity by enhancing exercise self-efficacy and improving perceptions of barriers to exercise benefits. A chain mediation pathway "social support → exercise self-efficacy → perception of barriers to exercise benefits → physical activity "was identified.

**Trial registration:**

The study protocol was approved by the Ethics Committee of Xi’an Jiaotong University (approval number: 20211575).

## Introduction

Maintenance hemodialysis is the primary treatment modality for patients with end-stage renal disease [1]. However, long-term dialysis therapy exerts persistent adverse effects on patients’ physiological status and quality of life. Among these adverse consequences, low levels of physical activity represent a prevalent and urgent issue among hemodialysis patients [2]. Existing evidence from studies focusing specifically on hemodialysis populations has confirmed that reduced physical activity is a key modifiable risk factor associated with sarcopenia, elevated risk of cardiovascular events, and increased all-cause mortality among hemodialysis patients [3–5]. Therefore, enhancing patients’ physical activity levels has become a critical intervention target for improving clinical outcomes and quality of life, and remains an important focus of clinical nephrology nursing research.

Social support—a vital external resource facilitating the adoption and maintenance of healthy behaviors—has been demonstrated to correlate with physical activity levels in patients with various chronic diseases [6–8].Research has shown that social support is significantly positively associated with physical activity levels—measured by daily total step count—among hemodialysis patients [9, 10]. However, the psychological mechanisms underlying how social support influences exercise behavior in this population remain unclear.

Exercise self-efficacy refers to an individual’s subjective confidence in their ability to maintain regular physical activity [11] ; it serves as a core intrinsic motivator for initiating and sustaining exercise behavior and is shaped through vicarious experiences and verbal persuasion [11]. For hemodialysis patients, the development of exercise self-efficacy can draw on critical external resources provided by social support [12]: successful exercise experiences of fellow patients offer vicarious learning opportunities, while exercise-related professional guidance and affirmation from healthcare providers constitute sources of verbal persuasion. Social support, as an external environmental factor, ultimately facilitates behavioral change by influencing individuals’ cognitive and psychological processes [13]. Specifically, social support enhances exercise self-efficacy among patients undergoing hemodialysis, thereby strengthening their confidence in engaging in physical activity and subsequently promoting higher levels of physical activity [14]. Based on this reasoning, the present study hypothesizes that exercise self-efficacy mediates the relationship between social support and physical activity level in hemodialysis patients.

Perceived barriers to exercise benefits refer to an individual’s subjective evaluation of both the value of exercise and the obstacles to its implementation [15]. According to the Health Belief Model, an individual’s decision to adopt a health behavior depends on their perception of disease threat and their comparative assessment of the perceived benefits and barriers associated with that behavior [16].Optimization of perceived benefits and barriers to exercise can be achieved through social support—a key external resource [17]. As an important external resource, social support influences hemodialysis patients’ exercise-related cognition along two dimensions: perceived benefits and perceived barriers. Specifically, it strengthens patients’ recognition of the therapeutic value of exercise while reducing their fear of exercise-related barriers, thereby diminishing resistance to behavioral enactment. Accordingly, this study hypothesizes that perceived benefits and barriers to exercise mediates the relationship between social support and physical activity levels among hemodialysis patients.

Self-Determination Theory (SDT) provides theoretical support for the above hypotheses. SDT posits that external environmental factors—such as social support—promote the development of intrinsic motivation for health behaviors by satisfying individuals’ psychological needs for autonomy, competence, and relatedness, thereby facilitating the adoption and maintenance of health behaviors [18].Social support can directly foster a sense of belonging among hemodialysis patients, thereby fulfilling their relatedness need; it can also facilitate the internalization of external support into intrinsic behavioral motivation through a progressive psychological process. By means of vicarious experiences and verbal persuasion, social support enhances patients’ exercise self-efficacy—instilling in them confidence in their ability to engage in regular physical activity—and thus satisfies their competence need. Once patients develop this confidence in exercising, they evaluate the benefits of physical activity and potential barriers to its implementation more positively, leading to heightened perception of exercise benefits and reduced concerns about exercise barriers. This cognitive shift reinforces patients’ autonomous motivation to engage in physical activity, satisfying their autonomy need and ultimately promoting an increase in their level of physical activity. Therefore, this study proposes a chain mediation pathway in this population: social support → exercise self-efficacy → perceived benefits and barriers to exercise → physical activity level. Therefore, the primary purpose of this study is to examine the chain mediating effects of exercise self-efficacy and perceived barriers to exercise benefits on the relationship between social support and physical activity levels among maintenance hemodialysis patients.

## Methods

### Participants

This study was a cross-sectional observational study conducted among hemodialysis patients at four tertiary hospitals in Xi’an City from September 2021 to February 2022. Inclusion criteria were as follows: ① receiving regular hemodialysis treatment (five to six sessions per two weeks); ② undergoing hemodialysis for at least three months; and ③ providing written informed consent. Exclusion criteria were as follows: ① cognitive impairment precluding cooperation with the survey; and ② severe physical mobility limitations preventing participation in exercise testing. Based on sample size calculations, 460 participants were enrolled. The study protocol was approved by the Ethics Committee of Xi’an Jiaotong University (approval number: 20211575). Participants provided written informed consent, and study procedures adhered to the ethical principles outlined in the Declaration of Helsinki.

### Measures

#### Variables

Study variables included sex, age, educational level, marital status, employment status, income, source of medical expenses, medical expenditure, etiology, duration of hemodialysis, and hemodialysis frequency.

#### Social support rating scale

Patients’ social support status was assessed using the Social Support Rating Scale developed by Xiao Shuiyuan [19]. The scale comprises 10 items, grouped into three dimensions: objective support (Items 2, 6, and 7), subjective support (Items 1, 3, 4, and 5), and utilization of social support (Items 8, 9, and 10). Total scores range from 12 to 66; scores ≤ 22 indicate low social support, 23–44 indicate moderate social support, and 45–66 indicate high social support. The Cronbach’s alpha coefficient of the scale in this measurement was 0.862, suggesting good reliability and validity [20].

#### Self-Efficacy for Exercise(SEE)

This study employed the Bandura Exercise Self-Efficacy Scale for measurement. The scale comprises 18 items assessing an individual’s confidence in engaging in regular physical activity under various conditions, including environmental or social influences, competing demands, and internal sensations. Responses are scored on a 10-point scale ranging from 0 to 100, with higher scores indicating greater exercise self-efficacy. Shin et al. administered this scale to Korean adults with chronic diseases and conducted a psychometric evaluation, reporting a Cronbach’s alpha coefficient of 0.94, a two-week test–retest reliability of 0.77, and confirmatory factor analysis supporting adequate construct validity [21]. Tung et al. translated the scale into Chinese; cross-translation and expert panel review confirmed its sound construct validity, with a Cronbach’s alpha coefficient of 0.942 [22].

### Dialysis Patient-Perceived Exercise Benefits and Barriers Scale(DPEBBS)

The DPEBBS is a self-administered questionnaire designed by Zheng et al. specifically for dialysis patients. This scale is intended to assess patients’ perceptions or awareness of the benefits of physical activity and the barriers hindering their engagement in exercise [23]. The scale comprises 24 items and two open-ended questions, with 12 items addressing perceived benefits of exercise and 12 addressing perceived barriers to exercise. Scoring follows a 4-point Likert scale. Items measuring exercise benefits are scored in the positive direction, whereas barrier items are scored in the reverse direction; the sum of scores from both categories constitutes the total scale score. The total score ranges from 24 to 96, with higher scores indicating greater perceived benefits of exercise and lower perceived barriers to exercise. The Cronbach’s alpha coefficient for the total scale score is 0.857, indicating good internal consistency reliability.

### Daily step count

Step counts were recorded using pedometers worn by study participants. In this study, OMRON-HJ321 electronic pedometers (OMRON) were used; participants wore the devices on their waist for seven consecutive days, removing them only during sleeping, bathing, or swimming [24]. The step-counting accuracy of this pedometer across varying walking speeds is ± 3%, making it one of the most accurate electronic pedometers reported in the literature to date. Correlation coefficients between step counts obtained with this model and those measured by the CSA (Computer Science and Applications, Inc.) accelerometer and Tritrac accelerometer range from 0.80 to 0.9307. Prior to data collection, all pedometers underwent pretesting to verify sensitivity. Researchers walked exactly 100 steps while correctly wearing the pedometer; the displayed count fell within the acceptable range of 100 ± 3 steps. Pedometers used in this study displayed step counts within ± 3 steps of the actual 100 steps walked by the researchers.

### Data collection

In this study, four tertiary hospitals in Xi’an were selected for hemodialysis treatment. The purpose, methodology, and anonymity of the study were explained to the subjects, and the patients who signed the informed consent form agreed to complete the questionnaire and wear a pedometer for 7 consecutive days. The online survey used the questionnaire star platform (WJX. CN) to collect electronic questionnaires. The system sets up a compulsory answering function to ensure there are no missing items. For those who do not have mobile phones or have difficulty filling in independently, the researchers assist in completing the questionnaire. A total of 468 questionnaires were distributed, and 460 valid questionnaires were collected, yielding a response rate of 98.3%. Data collection was conducted between September 2021 and February 2022.

### Data analysis

Independent samples t-tests and one-way analysis of variance (ANOVA) were used to analyze differences. Pearson correlation analysis was employed to examine the relationships among social support, exercise self-efficacy, barriers to exercise benefits, and total step count. Model 6 of the PROCESS v4.1 macro developed by Hayes et al. was used to test for chained mediation. The mediation effects were tested using the Bootstrap method, and the degree of common method bias was assessed using Harman’s one-way test. The analysis yielded 16 principal components, with the first component explaining 20.547% of the variance. Because this value is below 40%, it can be concluded that the data in this study do not exhibit significant common method bias. All statistical tests were two-tailed, and the significance level was set at α = 0.05.

### Ethical consideration

The study was conducted in accordance with the ethical principles of the Declaration of Helsinki. The study protocol was approved by the Ethics Committee of Xi’an Jiaotong University (Approval No.: 20211575). This approval ensured that all participants signed a written informed consent form prior to participating in the study and that special protective measures were implemented for individuals with cognitive impairments.

## Results

### Descriptive statistics and correlation analysis

Study participants were predominantly under 60 years old (73.3%), with males accounting for 66.7%. The frequency of hemodialysis was slightly higher at 5 sessions per 2 weeks (66.7%). Regarding the source of medical expenses, the vast majority relied on health insurance (96.3%). Regarding hemodialysis-related factors, the majority had been on hemodialysis for 36 months or longer (54.6%). In terms of etiology, unknown causes were the most common (27.4%). See Table 1 for other baseline characteristics.

**Table 1:** Demographic characteristics of subjects

Variable	Category	Frequency *n*	Percentage %
Gender	Male	307	66.7
	Female	153	33.3
Age	< 60 years old	337	73.3
	≥ 60 years old	123	26.7
Educational Level	Elementary school	13	2.8
	Junior high school	84	18.3
	High School	138	30
	College or Higher	225	48.9
Marital Status	Unmarried	61	13.3
	Married	370	80.4
	Divorced	12	2.6
	Widowed	17	3.7
Employment Status	Full-time	73	15.9
	Part-time	76	16.5
	Retired	151	32.8
	Unemployed/Job Seeker	160	34.8
Income	≥ 5,000 yuan	257	55.9
	<5,000 yuan	203	44.1
Source of Medical Expenses	Fully Self-Pay	9	2
	Health Insurance	443	96.3
	Fully government-funded	8	1.7
Medical Expenditures	<2000 yuan	194	42.2
	≥ 2000 yuan	266	57.8
Cause of Illness	Diabetic nephropathy	102	22.2
	Glomerulonephritis	102	22.2
	Hypertensive nephropathy	72	15.7
	Other	58	12.6
	Unknown cause	126	27.4
Duration of hemodialysis	<36 months	209	45.4
	≥ 36 months	251	54.6
Hemodialysis frequency	3 times/week	153	33.3
	5 times/2 weeks	307	66.7

### Participants’ social support, exercise self-efficacy, barriers to exercise benefits, and total scores for barriers to exercise benefits

The participants’ total social support score was 38.54 ± 10.75. Regarding exercise-related variables, the exercise self-efficacy score was 804.31 ± 393.79, with relatively large individual variations; the score for barriers to exercise benefits was 68.02 ± 9.18. Regarding physical activity levels, the total number of steps was 5221.89 ± 3156.10 (Table 2).

**Table 2:** Participants’ social support, exercise self-efficacy, barriers to exercise, and total score for barriers to exercise

Variables	Mean ± standard deviation
Social support	38.54 ± 10.75
Objective support	12.73 ± 3.94
Subjective support	20.44 ± 5.97
Social support utilization	5.37 ± 2.42
Physical activity self-efficacy	804.31 ± 393.79
Barriers to physical activity	68.02 ± 9.18
Total steps	5221.89 ± 3156.10

### Correlation analysis among participants’ social support, exercise self-efficacy, perceived barriers to exercise benefits, and total steps

Pearson correlation analysis revealed that, among study participants, social support was moderately positively correlated with exercise self-efficacy (*r* = 0.456, *P* < 0.01), and social support was also significantly positively correlated with perceived barriers to exercise benefits (*r* = 0.351, *P* < 0.01). Exercise self-efficacy and perceived barriers to exercise benefits showed a strong positive correlation (*r* = 0.507, *P* < 0.01), and total step count was significantly positively correlated with social support (*r* = 0.289, *P* < 0.01), exercise self-efficacy (*r* = 0.329, *P* < 0.01), and perceived barriers to exercise benefits (*r* = 0.305, *P* < 0.01). The correlation coefficients for each variable ranged from 0.289 to 0.507, all reaching statistical significance, as shown in Table 3.

**Table 3:** Correlation analysis

	Social Support	Social Support	Social Support	Social Support
Social Support	1			
Physical Activity Self-Efficacy	0.456**	1		
Barriers to Physical Activity	0.351**	0.507**	1	
Total Steps	0.289**	0.329**	0.305**	1

**, *P*<0.01

### A chain mediation analysis of the relationship between participants’ social support and physical activity, via exercise self-efficacy and perceived barriers to exercise benefits

#### Common method deviation test

To control the influence of common method bias on study results, Harman’s one-factor test to extract a total of 16 principal components was employed. The first principal component explained 20.547% of the variance, which is below the 40% threshold generally recommended in the literature [25]; therefore, the data in this study do not exhibit significant common method bias.

#### Chain mediating model and its multiple linear hierarchical regression analysis

all continuous variables were standardized. Model 6 in the PROCESS v4.1 macro developed by Hayes et al. [26] was used to examine the mediating effects of exercise self-efficacy and barriers to exercise benefits on the relationship between social support and total step count. Mediating effects were tested using stepwise regression; the results are presented in Table 4.

**Table 4:** Testing of mediator models

Variables	Model 1		Model 2		Model 3		Model 4	
	Total Steps		Exercise Self-Efficacy		Barriers to Exercise		Total Steps	
	β	t	β	t	β	t	β	t
Social support	0.289	6.457***	0.456	10.957***	0.151	3.375**	0.151	3.075**
Physical activity self-efficacy	—	—	—	—	0.438	9.791***	0.178	3.343**
Barriers to physical activity	—	—	—	—	—	—	0.162	3.196 **
R	0.289		0.456		0.524		0.389	
R2	0.083		0.208		0.275		0.152	
F	41.687***		120.058***		86.689***		27.162***	

Model 1 shows that social support has a significant positive effect on total steps (β = 0.289, t = 6.457, *P* < 0.001), indicating that the overall effect of social support on total steps is significant (*R* = 0.289, R² = 0.083, F = 41.687, *P* < 0.001)

Model 2 shows that social support has a significant positive effect on exercise self-efficacy (β = 0.456, t = 10.957, *P* < 0.001)

Model 3 shows that social support has a significant positive effect on the mediating variable, barriers to exercise benefits (β = 0.151, t = 3.375, *P* < 0.01), and that exercise self-efficacy has a significant positive effect on barriers to exercise benefits (β = 0.438, t = 9.791, *P* < 0.001), indicating that the first half of all three mediating pathways reached statistical significance (*R* = 0.524, R² = 0.275, F = 86.689, *P* < 0.001)

Model 4 shows that, after including exercise self-efficacy and barriers to exercise benefits, social support still has a significant positive effect on total steps (β = 0.151, t = 3.075, *P* < 0.01), and exercise self-efficacy (β = 0.178, t = 3.343, *P* < 0.01) and barriers to exercise benefits (β = 0.162, t = 3.196, *P* < 0.01) also had significant positive effects on total step count, suggesting that exercise self-efficacy and perceived benefits of exercise partially mediated the relationship between social support and total step count (*R* = 0.389, R² = 0.152, F = 27.162, *P* < 0.001)

*P<0.05, **P<0.01, ***P<0.001

### Bootstrap-based testing of mediating effects

**Fig. 1:**
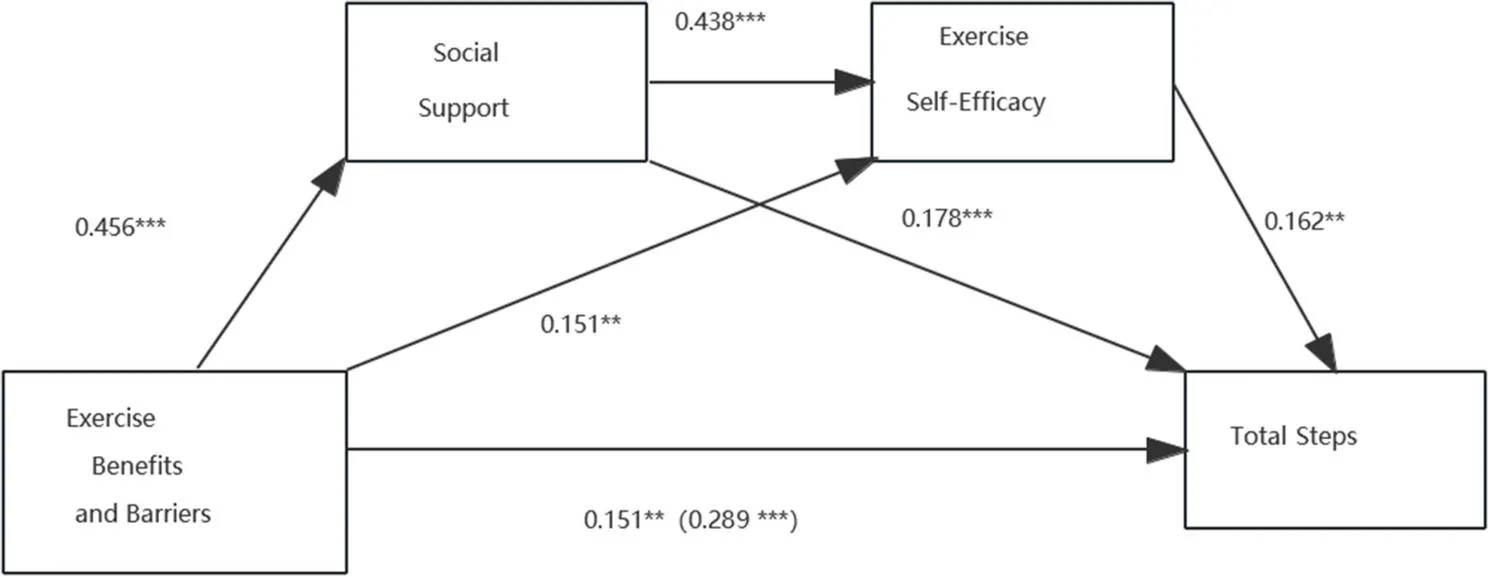
Chain mediating model diagram of social support and total steps between exercise self-efficacy and exercise benefits and barriers. Note: **P < 0.01

With social support as the independent variable and physical activity as the dependent variable, this study analyzed the mediating roles of exercise self-efficacy and barriers to exercise benefits. A structural equation model was established, and the mediating effects were tested using the Bootstrap method. The results indicated that the model fit well (Fig. 1). The total effect size of social support on total steps was 0.289 (SE = 0.045, 95% CI [0.201, 0.377]). The direct effect size was 0.151 (SE = 0.063, 95% CI [0.030, 0.274]), accounting for 52.25% of the total effect. The total indirect effect was 0.138 (SE = 0.032, 95% CI [0.078, 0.204]), accounting for 47.78% of the total effect.

The effect analyses for the three specific mediating pathways are shown in Table 5. The indirect effect value for Path 1 (social support → exercise self-efficacy → total steps) was 0.081 (SE = 0.029, 95% CI [0.025, 0.141]), accounting for 28.12% of the total effect, making it the primary mediating pathway. The indirect effect of Path 2 (social support → perceived exercise benefits/barriers → total steps) was 0.025 (SE = 0.011, 95% CI [0.007, 0.049]), accounting for 8.48%. The chain mediation effect for Path 3 (social Support → exercise Self-Efficacy → perceived barriers to exercise benefits → total daily Steps) was 0.032 (SE = 0.012, 95% CI [0.012, 0.058]), accounting for 11.18% of the total effect. None of the 95% confidence intervals included 0, indicating that the mediation effects were significant.

**Table 5:** Testing for mediating effects using bootstrap methods

Path of influence Effect	Path of influence Effect	SE	95%Lower	95%Upper	percentage
Total effect	0.289	0.045	0.201	0.377	
Direct effect	0.151	0.063	0.030	0.274	52.25%
Total indirect effect	0.138	0.032	0.078	0.204	47.78%
ind1	0.081	0.029	0.025	0.141	28.12%
ind2	0.025	0.011	0.007	0.049	8.48%
ind3	0.032	0.012	0.012	0.058	11.18%

Ind1: Social support → Exercise self-efficacy → Total steps

Ind2: Social support → Barriers to exercise benefits → Total steps

Ind3: Social support → Exercise self-efficacy → Barriers to exercise benefits → Total steps

## Discussion

Based on self-determination theory, this study examined the psychological mechanisms through which social support influences physical activity among hemodialysis patients. The results confirmed that social support not only has a direct positive effect on the physical activity levels of hemodialysis patients but also exerts an indirect effect through the mediating roles of exercise self-efficacy and perceived barriers to exercise benefits. Additionally, a chain of mediating pathways was identified: social support → exercise self-efficacy → perceived barriers to exercise benefits → physical activity levels. Overall, the direct and indirect effects account for a similar proportion of the total effect of social support on physical activity levels, indicating that the direct promotion of external support and its transformation through internal psychological mechanisms are equally important in promoting patients’ physical activity.

This study confirms that exercise self-efficacy plays a significant partial mediating role between social support and physical activity levels in hemodialysis patients, accounting for 28.12% of the total effect. This finding reveals how social support translates into specific physical activity behaviors: social support first acts on the patient’s psychological level, enhancing their core belief in exercise self-efficacy. Exercise self-efficacy, as an individual’s belief in their ability to maintain regular exercise, directly determines their behavioral motivation, effort, and persistence [27]. Hemodialysis patients with high exercise self-efficacy are better able to proactively activate behavioral regulation mechanisms when facing exercise barriers such as fatigue and discomfort, reassessing exercise difficulty and mobilizing psychological resources [28]. Through positive self-talk and recollecting past successes, these patients transform negative feelings into actionable coping strategies. This belief-based support enables hemodialysis patients to set realistic exercise goals and continuously accumulate successful experiences during the goal-achievement process [29]. Such reinforcement of self-efficacy reinforces their sense of control over health behaviors, enhances their psychological resilience in overcoming challenges during disease management, and ultimately transforms internal confidence into sustained and consistent physical activity. This pattern of self-efficacy beliefs driving behavioral practice is consistent with findings from studies on other chronic disease populations [30, 31]. For example, in a study of stroke patients, Jiang et al. demonstrated that rehabilitation self-efficacy positively influences patients’ expectations regarding exercise outcomes, indicating that patients with stronger self-efficacy also perceive the benefits of physical activity more strongly [32]. In summary, exercise self-efficacy serves as a crucial psychological mediator linking external social support to individual health behaviors.

The results of this study confirm the perception of barriers to exercise benefits partially mediates the relationship between social support and physical activity levels in hemodialysis patients, with a mediation effect of 8.48%. This finding suggests social support helps patients evaluate the pros and cons of exercise with a more positive attitude; this cognitive shift aligns with the need for autonomy in Self-Determination Theory. In contrast, Ma et al., in a study of heart failure patients, found psychological resilience mediated 28.5% of the relationship between perceived social support and physical activity levels [33]. Comparing the Comparing the findings of the present study with those of Ma et al., the mediating effect of psychological resilience was significantly higher than that of the perception of exercise benefits and barriers. Although both studies confirmed the existence of psychological mediating mechanisms between social support and physical activity levels, and both mediating variables were associated with social support and physical activity levels, there were clear differences in the mediating effects and mechanisms of action. In this study, the perception of barriers to exercise benefits represents patients’ assessment of the benefits and difficulties associated with exercise and serves as a positive mediating variable. Social support positively promotes increased physical activity levels by enhancing patients’ perception of exercise benefits and reducing their concerns about exercise barriers. In contrast, psychological resilience in the study by Ma et al. [33] refers to a trait that enables patients to maintain emotional stability and adaptability when facing the stress of illness. Social support enhances patients’ psychological resilience, thereby increasing their stress tolerance and persistence, which in turn promotes higher levels of physical activity. Although the two studies differ fundamentally in the nature of their mediating variables and the logical mechanisms through which they influence physical activity, they both corroborate the core tenet of Self-Determination Theory: external environmental factors must influence an individual’s internal cognitive and psychological processes to be effectively translated into specific health behaviors.

The current study also found that exercise self-efficacy and the perception of barriers to exercise benefits play a chained mediating role between social support and physical activity levels, accounting for 11.18% of the mediating effect. Specifically, social support first enhances patients’ exercise self-efficacy; this increased confidence further encourages them to evaluate the value of exercise and the associated barriers more positively, ultimately leading to an increase in physical activity levels. This progressive psychological mechanism aligns with the chain mediation logic of “psychological response → gratitude → sense of life meaning → coping ability” identified by Chen et al. [33], both demonstrating progressive pathways among psychological variables. In this study, confident patients were more likely to actively attempt exercise; during this process, they personally experienced the positive benefits of exercise, thereby reinforcing their perception of its benefits. Simultaneously, they gained successful experiences in overcoming barriers, which in turn reduced their anxiety regarding these barriers. This pathway formed a virtuous cycle suggesting that clinical intervention strategies should be comprehensive and systematic. In the initial phase, the focus should be on establishing a supportive social environment to enhance self-efficacy; in the intermediate phase, patients should be guided to engage in exercise under safe conditions to gain positive experiences; and in the later phase, these experiences should be reinforced to consolidate positive perceptions of exercise, thereby establishing long-term, self-directed exercise habits. From the perspective of self-determination theory, the core of this cycle lies in external social support progressively fulfilling internal psychological needs. Social support meets patients’ competence needs through professional guidance and experience sharing, laying the foundation for confidence in exercise; positive feedback during exercise practice encourages patients to actively evaluate the value of exercise, satisfying their need for autonomy; while support such as family companionship and encouragement from fellow patients continuously meets relational needs, providing emotional support.

This study found the core theoretical basis for the impact of social support on physical activity among hemodialysis patients is Self-Determination Theory: external support must stimulate and satisfy individuals’ intrinsic psychological needs to facilitate changes in exercise behavior. Social support first enhances patients’ exercise self-efficacy, satisfying their need for competence. This subjective confidence in one’s own exercise ability serves as the key motivator for patients to overcome exercise barriers and initiate exercise behavior [30]. Once patients establish confidence in their exercise capabilities, they reassess the benefits and barriers of exercise from a more positive perspective, optimizing their perception of exercise benefits and barriers, and further satisfying their need for autonomy [30]. Finally, the combined effect of fulfilled competence and autonomy transforms into intrinsic motivation for patients to engage in physical activity, driving the formation of more enduring and stable exercise behaviors. The chain of mediating pathways identified in this study—social support → exercise self-efficacy → perception of exercise benefits and barriers → physical activity—serves as concrete evidence of Self-Determination Theory in this population. This pathway clearly illustrates how external social support facilitates a transition from external behavioral, prompting internal motivation by sequentially fulfilling individuals’ needs for competence and autonomy, thereby prompting patients to shift from passively receiving support to actively engaging in health-promoting behaviors.

This study has certain limitations that require further refinement in future research. First, as a cross-sectional study, it can only identify correlations and mediating effects among variables but cannot establish causal relationships. Second, the participants in this study were exclusively recruited from four tertiary hospitals in Xi’an. Restricted by the hospital tier, the sample has limited representativeness, and further research is required to extend the findings to hemodialysis patients in primary hospital dialysis centers. In addition, most participants were under 60 years old, and their average daily step count was relatively high. Although this age distribution aligns with the younger trend of hemodialysis patients in China, the results cannot be readily generalized to elderly hemodialysis patients in Western countries or those receiving dialysis in primary medical institutions. Third, physical activity levels were measured solely by daily step count, without incorporating indicators such as exercise duration or intensity, resulting in an incomplete assessment of physical activity. Fourth, data were collected via questionnaires. Although the Harman single-factor test confirmed no significant common method bias, some subjective response bias may exist.

## Conclusion

This study, which examined 460 hemodialysis patients at a tertiary hospital in Xi’an, yielded the following results: social support positively influences the daily physical activity levels of hemodialysis patients, with exercise self-efficacy and perceived barriers to exercise benefits mediating this relationship. Additionally, a chain mediation effect was observed: social support → exercise self-efficacy → perceived barriers to exercise benefits → total daily step count. This conclusion confirms that social support promotes physical activity by altering patients’ exercise self-efficacy and perceived barriers to exercise benefits.

Based on these findings, this study offers multifaceted intervention strategies for clinical practice. First, healthcare professionals should establish robust social support systems for patients, provide professional exercise guidance, instruct family members to offer emotional and practical support during dialysis sessions, and create platforms for peer-to-peer communication to facilitate the exchange of experiences, thereby providing patients with diverse forms of social support. Second, healthcare professionals can develop personalized exercise plans for patients to effectively enhance their sense of achievement and strengthen their confidence in their own exercise capabilities. Finally, they should improve patients’ perceptions of the benefits and barriers to exercise by conducting targeted health education to explain the positive effects of light physical activity on dialysis treatment and physical function. At the same time, they should help patients overcome exercise barriers—such as by planning exercise schedules reasonably and adjusting exercise methods—so that patients can truly feel that these barriers are surmountable.

Future research could build upon the limitations of this study by adopting a cohort study design to further clarify the causal relationships among variables; expanding the sample scope to include hemodialysis patients from different regions and hospitals of varying levels to enhance the representativeness of the findings; and refining the assessment indicators for physical activity to include a comprehensive evaluation of exercise duration, intensity, and type. Furthermore, based on the findings of this study, specific intervention protocols could be designed and tested through randomized controlled trials to validate their efficacy and feasibility, thereby providing more compelling intervention strategies to enhance physical activity levels among hemodialysis patients.

Overall, this study provides new theoretical foundations and practical guidance for physical activity interventions among hemodialysis patients, confirming the mediating roles of exercise self-efficacy and perceived barriers to exercise benefits in the relationship between social support and physical activity. In clinical practice, both external social support and internal psychological support must be addressed simultaneously to more effectively improve the physical activity levels of hemodialysis patients, thereby enhancing their dialysis outcomes and physical function.

## Data Availability

The datasets generated and analysed during the current study are not publicly available but are available from the corresponding author on reasonable request.

## Abbreviations

SSRS: Social Support Rating Scale
SEE: Self-Efficacy for Exercise Scale
DPEBBS: Dialysis Patient-perceived Exercise Benefits and Barriers Scale
SDT: Self-Determination Theory
NOVA: Analysis of Variance
